# Beyond Charcot’s Triad: Occult Choledocholithiasis Unmasked by Polymicrobial Sepsis and Multifocal Pyogenic Liver Abscesses

**DOI:** 10.7759/cureus.117991

**Published:** 2026-10-15

**Authors:** Hunter Wayland, Tanner Petty, Caleb Harkins, David R Janese

**Affiliations:** 1 Emergency Medicine/Family Medicine, Louisiana State University Health Sciences Center Shreveport (LSUHSC-S), Shreveport, USA; 2 Intensive Care, Willis Knighton, Shreveport, USA

**Keywords:** acute cholangitis, biliary obstruction, extended-spectrum beta-lactamase, polymicrobial bacteremia, pyogenic liver abscess, rural hospital

## Abstract

Suspected choledocholithiasis/biliary obstruction complicated by ascending cholangitis with pyogenic liver abscesses can rapidly progress to bacteremia, sepsis, and multiorgan dysfunction including distributive shock. Recognition may be delayed when patients lack characteristic diagnostic features, and patients of advanced age or with other comorbidities may present with nonspecific neurologic and metabolic abnormalities that obscure the underlying source. This report describes an atypical case of occult complicated biliary obstruction presenting as refractory polymicrobial sepsis in a 64-year-old woman with type 2 diabetes mellitus and multiple other chronic medical conditions, examining the evolution of clinical findings that ultimately prompted escalation of care including transfer for definitive management. Occult hepatobiliary infection should be considered early in patients with persistent polymicrobial and/or extended-spectrum beta lactamase (ESBL) bacteremia and evolving cholestatic abnormalities, even when Charcot’s triad is absent. Early contrast-enhanced imaging and timely biliary decompression, with abscess drainage when indicated, are essential as antimicrobial therapy alone cannot achieve definitive source control.

## Introduction

Choledocholithiasis - the presence of gallstones within the common bile duct (CBD) - is a well-established cause of biliary obstruction and ascending cholangitis [1]. When biliary obstruction occurs, bile stasis promotes bacterial overgrowth within the biliary tree, and elevated intraluminal pressure disrupts tight junctions, driving bacteria into the hepatic vasculature and perihepatic lymphatics, leading to bacteremia [2]. Biliary organisms are identified in up to 90% of patients with choledocholithiasis accompanied by jaundice [2]. Pockets of pus can subsequently develop in the liver as a downstream complication of this cascade, a condition known as pyogenic liver abscess (PLA).

PLA is a serious intra-abdominal infection most commonly arising from biliary tract disease, including choledocholithiasis and biliary strictures, and frequently involves polymicrobial enteric organisms including Escherichia coli, Streptococcus spp., and Klebsiella pneumoniae [3]. Importantly, the classic triad of fever, jaundice, and right upper-quadrant pain is frequently absent; instead, PLA often presents with nonspecific constitutional symptoms such as fever, malaise, and weight loss, making it one of the most diagnostically challenging abdominal conditions [3,4]. Diabetes mellitus, present in 23%-58% of PLA patients across published series, is a major risk factor due to impaired neutrophil chemotaxis and phagocytic function in the setting of hyperglycemia [4-6]. Few reports describe PLA presenting solely as polymicrobial sepsis without Charcot’s triad in post-cholecystectomy patients.

This case demonstrates how atypical presentation combined with competing acute diagnoses and delayed recognition of the underlying biliary source produced near-fatal consequences.

## Case presentation

Patient background

A 64-year-old African American female with a past medical history significant for hypertension, type 2 diabetes mellitus (T2DM), prior transient ischemic attacks/cerebrovascular accidents (TIA/CVA), stage 3 chronic kidney disease (CKD) with baseline creatinine of 1.5-2.0 mg/dL, and long-term tobacco use (quit two weeks before admission) presented to the Emergency Department on day 1 after three days of bed rest, believing she had suffered a "small stroke." Family members noted that she was not acting like herself and was unable to complete activities of daily living independently. Home medications included losartan 100 mg daily, amlodipine 10 mg daily, glipizide 10 mg twice daily, hydralazine 25 mg twice daily, and doxazosin 2 mg twice daily. The patient had no known drug allergies.

Initial presentation

On arrival, the patient’s vital signs were as follows: BP 104/69 mmHg, HR 80 beats per minute, RR 16 breaths per minute, Temp 97.9 °F, and SpO2 100% on room air. Physical examination revealed an elderly woman who was alert and in no acute distress, without focal neurologic deficits, with lungs clear to auscultation bilaterally, and with a nontender and nondistended abdomen. Specifically, it was noted that there was no right upper quadrant tenderness on first examination or at any point later in her primary hospitalization. Initial laboratory investigations (Table 1) and an unremarkable CT head without contrast led to a differential diagnosis for encephalopathy that included urinary tract infection, metabolic acidosis, TIA/CVA, medication side effects, and derangements associated with CKD as potential contributors to the clinical picture. Laboratory data showed that the patient was acutely hypoglycemic at 30 mg/dL, which favored a metabolic encephalopathy.

**Table 1:** Initial laboratory results indicating systemic inflammation in the setting of suspected UTI/pyuria; metabolic derangements, including hypoglycemia, hyponatremia, and metabolic acidosis; and findings consistent with stage 3 chronic kidney disease (CKD3), including proteinuria, hypoalbuminemia, and anemia WBC, white blood cell; CO₂, carbon dioxide; ALP, alkaline phosphatase; AST, aspartate aminotransferase; UA, urinalysis; UTI, urinary tract infection; hpf, high-power field; dx, diagnosis

Test	Result	Adult female reference range	Significance/Context
WBC	17.3 x 10³/µL	3.5-10.5 x 10³/µL	Leukocytosis
Differential	93% neutrophils	40%-70% neutrophils	Neutrophilia
Hemoglobin	8.8 g/dL	12.0-15.5 g/dL	Anemia
Platelets	247 x 10³/µL	150-400 x 10³/µL	Normal initially
Glucose	30 mg/dL (on arrival)	60-99 mg/dL fasting	Critical hypoglycemia
Sodium	133 mmol/L	136-145 mmol/L	Hyponatremia
CO_2_	16 mmol/L	22-27 mmol/L	Metabolic acidosis
Creatinine	1.8 mg/dL (baseline)	0.6-1.1 mg/dL	Elevated w/dx CKD
Albumin	3.1 g/dL	3.5-5.0 g/dL	Hypoalbuminemia
Total bilirubin	0.9 mg/dL	0.1-1.2 mg/dL	Normal initially
ALP	164 U/L	40-130 U/L	Elevated w/dx CKD
AST	36 U/L	10-40 U/L	Normal initially
UA Leukocytes	WBC 50-100/hpf	WBC 0-5/hpf	Pyuria suspicious of UTI
UA protein	100 mg/dL	<30 mg/dL	Proteinuria w/dx CKD

The absence of clinical jaundice, right upper-quadrant pain, and hyperbilirubinemia - the hallmarks of biliary obstruction - meant that the hepatobiliary system was not initially suspected as the underlying infectious source. The patient was started on ceftriaxone for suspected UTI/pyuria coverage and was given IV fluids and supportive care. Blood cultures were obtained on the first day of admission, and CT of the chest/abdomen/pelvis without contrast (Figure 1) demonstrated no significant findings on the official read to correlate with the clinical picture.

**Figure 1:**
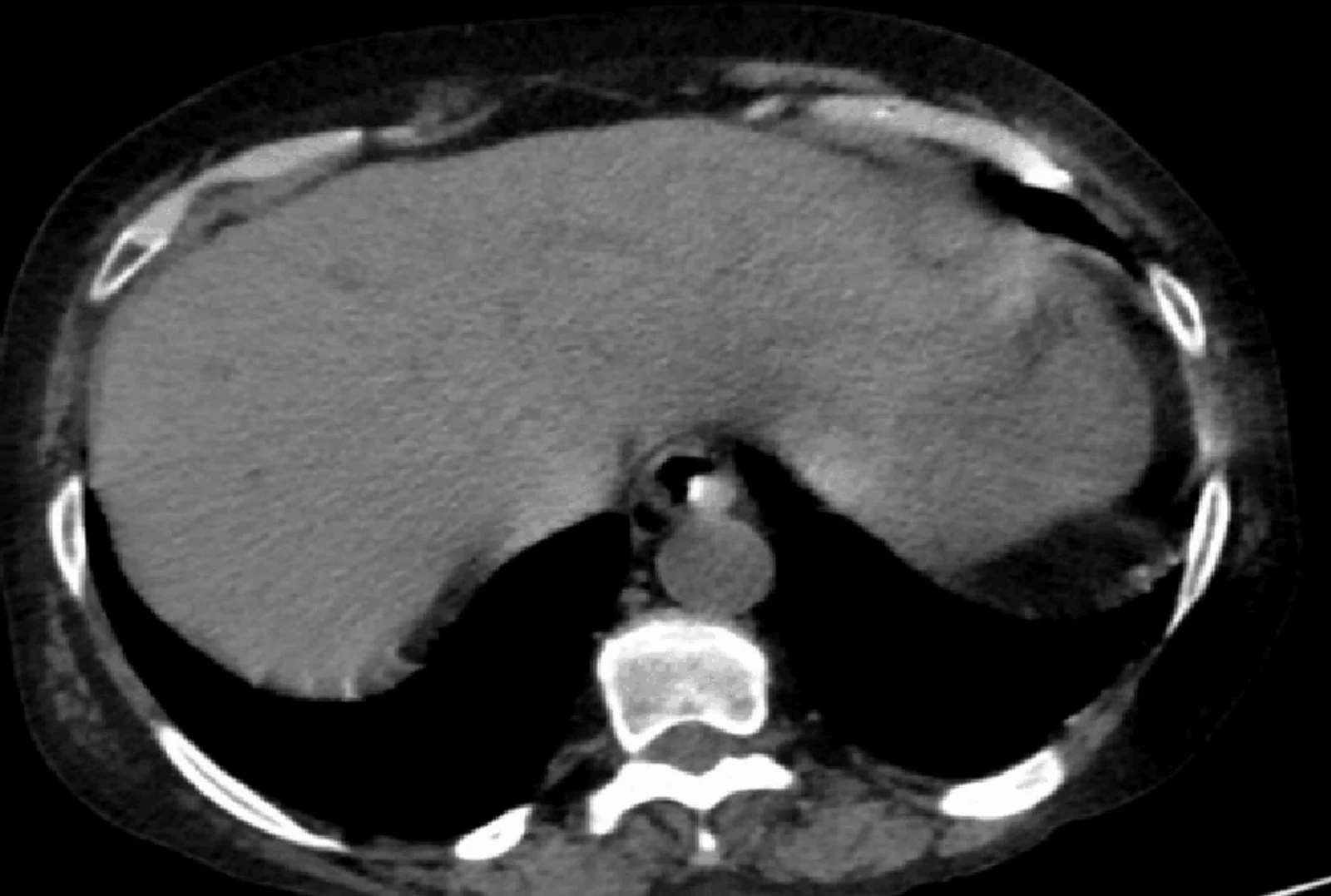
CT of the abdomen without contrast, obtained shortly after the patient’s presentation, showed no evidence of hepatobiliary involvement on the official radiology report.

Course of primary hospitalization

Despite appropriate treatment of the identified diagnoses, the patient failed to defervesce, and her leukocytosis progressively worsened. A respiratory viral PCR panel was negative for all tested pathogens, including SARS-CoV-2, influenza A/B, RSV, adenovirus, and common respiratory bacteria. MRI of the brain confirmed the absence of an acute stroke. On day 4, the patient's platelet count dropped precipitously from 247 to 16 × 10³/µL. This consumptive thrombocytopenia was one of the first signs that a more significant infectious process was driving the clinical picture. Platelet transfusions were initiated. Other laboratory indicators of clinical non-improvement included persistent, clinically significant leukocytosis, as well as ongoing low-grade fevers (Table 2).

**Table 2:** Trends in laboratory investigations during the patient’s primary hospitalization demonstrated persistent, initially non-improving leukocytosis and ongoing fevers, followed by a sharp increase in liver function tests. WBC, white blood cell; ALP, alkaline phosphatase; AST, aspartate aminotransferase.

Hospital day	1	2	3	4	5	6	7	8	Adult female reference range
WBC (x10³/µL)	15.3	17.3	18.5	22.5	15.8	-	-	-	3.5-10.5 x 10³/µL
T_max_ (°F)	97.9	101.6	101.0	100.3	100.8	-	-	-	96.8-100.4
Total bilirubin	0.9	-	-	-	0.7	1.2	1.3	4.8	0.1-1.2 mg/dL
ALP (U/L)	164	-	-	-	170	298	289	426	40-130 U/L
AST (U/L)	36	-	-	-	43	102	81	120	10-40 U/L

Blood cultures resulted with critical findings on days 2, 3, and 4 of admission, identifying polymicrobial bacteremia with three distinct organisms, including extended-spectrum beta-lactamase (ESBL)-producing Escherichia coli, a multidrug-resistant organism with resistance to ampicillin, ceftriaxone, and trimethoprim/sulfamethoxazole. The susceptibility profile of this ESBL MDRO E. coli strain included meropenem (MIC ≤ 0.25), piperacillin/tazobactam (MIC ≤ 4), gentamicin, ciprofloxacin, and levofloxacin. Other organisms identified in addition to E. coli were Enterococcus avium and a Streptococcus species that was not further speciated.

As delineated in Table 2, concomitant progressive worsening of liver markers at the one-week inpatient threshold indicated an obstructive and potentially parenchymal hepatobiliary process. This prompted reimaging with CT chest/abdomen/pelvis, this time with IV contrast, which revealed the underlying driver of the patient’s illness. The official radiology report noted hepatomegaly with multifocal hypodense fluid collections in the left hepatic lobe - the largest lesion in segment 3 measuring 5.0 × 3.6 cm - highly concerning for pyogenic hepatic abscesses, intrahepatic biliary dilatation with the CBD enlarged to 2.2 cm (normal < 8 mm), and coarse calcifications in the pancreatic head with pancreatic duct dilation to 7 mm, consistent with choledocholithiasis and/or chronic pancreatitis. Additional findings included small bilateral pleural effusions with bibasilar atelectasis, a small pericardial effusion, anasarca, and a surgically absent gallbladder (Figure 2).

**Figure 2:**
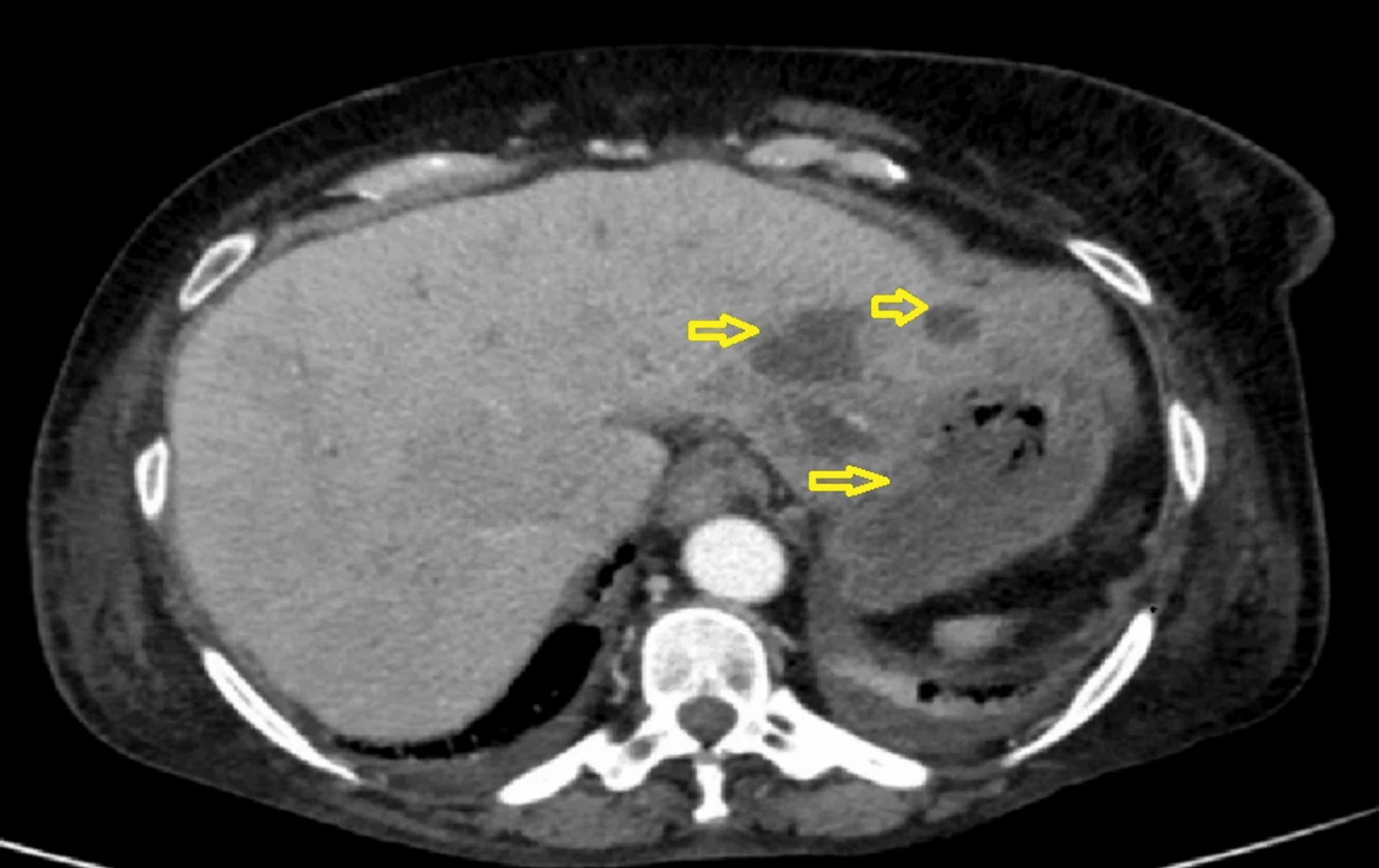
CT abdomen with contrast later in the primary hospitalization revealed a number of concerning findings, including evidence of complex hepatobiliary infection. Arrows indicate abscess formation.

Retrospective application of the Tokyo Guidelines 2018 (TG18) clarifies when the hepatobiliary source became objectively supportable. TG18 defines suspected acute cholangitis as at least one criterion for systemic inflammation (A) plus either cholestasis (B) or compatible imaging (C), and a definite diagnosis as at least one criterion from each of the A, B, and C domains (Table 3) [7].

**Table 3:** Temporal application of Tokyo Guidelines 2018 diagnostic and severity criteria. The patient did not meet objective cholangitis criteria at initial presentation, met criteria for suspected acute cholangitis as biochemical cholestasis emerged by day 6, and met criteria for definite Grade III acute cholangitis after day 8 imaging demonstrated biliary dilatation. WBC, white blood cell count; T_max_, maximum temperature; ALP, alkaline phosphatase; GGT, gamma-glutamyl transferase; AST, aspartate aminotransferase; ALT, alanine aminotransferase; ULN, upper limit of normal; CT, computed tomography; CBD, common bile duct

TG18 domain/status	Objective criterion	Patient findings and timing	Interpretation
A. Systemic inflammation	Fever >38 °C and/or WBC <4 or >10 x 10³/µL	Day 1 WBC 15.3-17.3 x 10³/µL; day 2 Tmax 101.6 °F (38.7 °C); day 4 WBC 22.5 x 10³/µL.	Met from admission
B. Cholestasis	Total bilirubin ≥2 mg/dL or ALP/GGT/AST/ALT >1.5 x ULN	Not met initially (bilirubin 0.9 mg/dL; ALP 164 U/L; AST 36 U/L). Day 6 ALP 298 U/L and AST 102 U/L exceeded 1.5 x ULN; day 8 bilirubin 4.8 mg/dL.	Met by day 6
C. Imaging	Biliary dilatation and/or imaging evidence of an obstructing etiology	Initial noncontrast CT: no hepatobiliary abnormality on official read. Day 8 contrast-enhanced CT: intrahepatic biliary dilatation and CBD 2.2 cm.	Met on day 8
Diagnostic classification	Suspected: A + (B or C). Definite: A + B + C.	Systemic inflammation preceded objective cholestasis; biochemical cholestasis emerged by day 6; biliary dilatation was demonstrated on day 8.	Suspected by day 6; definite by day 8
Severity	Grade III if dysfunction of any one specified organ/system is present; hematologic criterion: platelets <100 x 10³/µL.	Platelets fell from 247 to 16 x 10³/µL on day 4. Altered mental status was also present, although initially confounded by metabolic abnormalities.	Grade III (severe)

At presentation, the patient met the systemic inflammation domain by leukocytosis (WBC 15.3 - 17.3 × 10³/µL) but did not yet meet TG18 cholestasis thresholds: total bilirubin was 0.9 mg/dL, ALP was 164 U/L (1.26 times the upper limit of normal (ULN)), and AST was 36 U/L; the initial noncontrast CT also showed no biliary abnormality on official interpretation. Systemic inflammation became more overt on day 2 with fever to 101.6 °F (38.7 °C), while leukocytosis persisted through day 5. By day 6, biochemical cholestasis met the TG18 B-2 threshold, with ALP 298 U/L (2.29 times ULN) and AST 102 U/L (2.55 times ULN), supporting a suspected diagnosis of acute cholangitis in the setting of established systemic inflammation. On day 8, total bilirubin reached 4.8 mg/dL, and contrast-enhanced CT demonstrated intrahepatic biliary dilatation with CBD dilatation to 2.2 cm, thereby fulfilling the cholestasis and imaging domains and establishing a definite TG18 diagnosis [7].

Treatment

Antimicrobial therapy was escalated to meropenem, and MRCP was recommended for further characterization of the hepatobiliary abnormalities. Intravenous dextrose was administered for hypoglycemia but did not improve the encephalopathy. Given the marked common bile duct dilation, multifocal hepatic abscesses, and concern for an incompletely controlled biliary source, the patient was transferred to a higher-level facility with gastroenterology, interventional radiology, and surgical capabilities should biliary decompression or abscess drainage become necessary. Following transfer, however, she demonstrated substantial clinical improvement with intravenous antimicrobial therapy, including resolution of bacteremia. Because of this favorable response, no endoscopic, percutaneous, or operative intervention was ultimately performed.

## Discussion

The classical features of Charcot's triad (fever, jaundice, right upper quadrant pain) and Reynolds' pentad (Charcot's triad + hypotension + altered mental status) were largely absent throughout this patient's course. Several features complicated arrival at the final diagnosis: an atypical presentation and lack of development of hallmark features, competing alternative differential diagnoses that were more consistent with the presentation and appropriately dominated the clinical picture, unremarkable initial imaging, and prior cholecystectomy, which blunted the expectation of biliary findings in this context.

The disproportionate elevation of ALP over transaminases is the biochemical fingerprint of biliary obstruction rather than direct hepatocellular injury [4]. In this case, however, total bilirubin remained below the threshold of clinical jaundice (>2 to 3 mg/dL) until day 8, suggesting progressive subtotal or intermittent biliary obstruction from choledocholithiasis [1]. Contrast-enhanced CT findings confirmed the unifying pathophysiologic sequence: choledocholithiasis leading to biliary obstruction and ascending cholangitis, with subsequent hepatic parenchymal seeding via biliary canaliculi and portal circulation, causing the formation of pyogenic hepatic abscesses and ultimately polymicrobial bacteremia progressing to sepsis with significant end-organ dysfunction [2,3].

T2DM is among the most significant risk factors for PLA, present in 23-58% of cases across published series [5,6]. Impaired neutrophil function, altered biliary motility, and susceptibility to biliary infection all contribute to heightened risk in this population [8]. Notably, patients with newly diagnosed T2DM face a risk of PLA approximately 2.83- to 4-fold higher than that of non-diabetic counterparts in the first two years following diagnosis [5]. This patient's consistently elevated blood glucose levels (upwards of 331 mg/dL throughout admission) likely contributed to both her vulnerability to polymicrobial infection and the severity of her clinical course.

Biliary tract disease - including choledocholithiasis - is now the leading cause of PLA in the Western world, having surpassed appendicitis as the primary etiology [3]. Biliary bacteria are identified in 90% of patients with choledocholithiasis accompanied by jaundice; these bacteria ascend from the duodenum via the sphincter of Oddi, colonize the biliary tree, and are driven into the hepatic sinusoids and parenchyma by elevated intraluminal pressure, forming microabscesses that can coalesce into macroscopic collections [2]. In this case, development of abscesses measuring up to 5.0 cm - above the threshold at which percutaneous catheter drainage is strongly recommended - suggests an insidious and prolonged process predating hospitalization by days to weeks [3].

The absence of typical symptoms is a well-recognized hallmark of PLA, which often follows a subacute evolution that delays medical intervention until bacteremia or systemic complications have become evident [3,4]. E. coli is the most frequently isolated pathogen in biliary tract infections, and the identification of ESBL-producing E. coli bacteremia carries significant therapeutic implications [9]. The ESBL phenotype confers resistance to cephalosporins and penicillins despite apparent in vitro susceptibility. The landmark MERINO trial [10] demonstrated 30-day mortality of 12.3% with piperacillin/tazobactam versus 3.7% with meropenem for ESBL bloodstream infections - an absolute risk difference of 8.6% - establishing carbapenems as the Infectious Diseases Society of America (IDSA)-preferred definitive therapy for ESBL bacteremia outside the urinary tract [11].

Beyond ESBL-producing bacteremia, mixed Gram-negative and Gram-positive polymicrobial cholangitis carries a significantly longer hospitalization course and higher mortality rate than monomicrobial Gram-negative infections [12]. IDSA guidelines on intra-abdominal infections identify inadequate source control as a key determinant of mortality in patients with intra-abdominal sepsis and bacteremia [13]. This remains a central consideration in the management of acute cholangitis and pyogenic liver abscess, particularly when there is persistent biliary obstruction, ongoing bacteremia or sepsis despite appropriate antimicrobial therapy, or a large and technically drainable abscess. For PLA, the cornerstone of treatment is broad-spectrum IV antibiotics combined with percutaneous or surgical drainage, particularly for abscesses >3-5 cm in diameter [3]. ERCP is sometimes an alternative to percutaneous or open surgical approaches to relieve underlying biliary obstruction, without which ongoing bacterial hematogenous seeding perpetuates bacteremia regardless of the antibiotic regimen [2,13]. Each hour of delay in appropriate antibiotics independently increases the odds of death in patients with cholangitis-associated sepsis, and mortality risk triples when biliary decompression is postponed beyond 12 hours [14]. This timeline presents a challenging goal for the rural community practitioner, who may not have access to advanced imaging capabilities, interventional management options, or immediately available specialty staff to arrange them for patients with complex deep abdominal infections. Even making a diagnosis without access to dedicated endoscopic gastrointestinal (GI) services for visualization and culture of the biliary tract can be difficult, particularly in patients such as these who present with atypical and/or mixed clinical pictures. Importantly, however, not every patient ultimately requires an invasive procedure. In this case, the radiographic findings and persistent sepsis appropriately prompted transfer to a facility capable of biliary and percutaneous intervention, but the patient improved after escalation of antimicrobial therapy and cleared her bloodstream infection without ERCP, drainage, or surgery. Thus, the transfer provided access to definitive source-control options if needed, while subsequent clinical improvement allowed invasive intervention to be deferred.

Outcomes

Following transfer to a larger medical center with advanced interventional capabilities, the patient continued intravenous antimicrobial therapy and demonstrated progressive clinical improvement. Blood cultures subsequently cleared, and because she improved without evidence of ongoing uncontrolled sepsis, no endoscopic, percutaneous, or operative source-control procedure was ultimately performed. She was discharged home in stable condition. Her post-hospitalization course was later complicated by clinically significant non-Clostridioides difficile diarrhea and prerenal acute kidney injury related to severe dehydration.

## Conclusions

This case illustrates a clinically insidious presentation in which suspected choledocholithiasis or other biliary obstruction was associated with ascending cholangitis, multifocal pyogenic liver abscesses, polymicrobial ESBL bacteremia, and sepsis with multiorgan dysfunction despite the absence of classic biliary symptoms. The diagnostic course was prolonged by competing acute diagnoses, initially normal bilirubin, and nondiagnostic early noncontrast imaging. Contrast-enhanced cross-sectional imaging ultimately revealed the hepatobiliary source and prompted transfer to a center capable of ERCP, interventional drainage, and surgical management if required. Although invasive source control was considered appropriate given the imaging findings and severity of illness, the patient improved with intravenous antimicrobial therapy and did not ultimately require ERCP, abscess drainage, or surgery. This case therefore highlights both the importance of early recognition and access to source-control capability and the need to individualize invasive management according to the patient’s subsequent clinical response.
